# *Trapa natans L*. Extract Attenuates Inflammation and Oxidative Damage in Cisplatin-Induced Cardiotoxicity in Rats by Promoting M2 Macrophage Polarization

**DOI:** 10.1155/mi/6587305

**Published:** 2025-01-22

**Authors:** Vesna Matovic, Biljana Ljujic, Ivana Radojevic, Gorica Đelic, Marina Miletic Kovacevic, Suzana Zivanovic, Milos Papic, Nevena Milivojevic, Ivica Petrovic, Marina Gazdic Jankovic

**Affiliations:** ^1^Faculty of Medical Sciences, Department of Genetics, University of Kragujevac, Kragujevac, Serbia; ^2^Faculty of Medical Sciences, Center for Harm Reduction of Biological and Chemical Hazards, University of Kragujevac, Kragujevac, Serbia; ^3^Faculty of Science, Department of Biology and Ecology, University of Kragujevac, Radoja Domanovica 12, Kragujevac 34000, Serbia; ^4^Faculty of Medical Sciences, Department of Histology and Embryology, University of Kragujevac, Kragujevac, Serbia; ^5^Department of Dentistry, Faculty of Medical Sciences, University of Kragujevac, Kragujevac, Serbia; ^6^Laboratory for Bioengineering, Institute of Information Technologies Kragujevac, University of Kragujevac, Kragujevac, Serbia; ^7^Faculty of Medical Sciences, Department of Pathophysiology, University of Kragujevac, Kragujevac, Serbia

**Keywords:** cardiotoxicity, cisplatin, *Trapa natans* L. extract

## Abstract

**Background:** *Trapa natans* L. fruits and leaf extracts have a broad range of immunomodulatory, anti-inflammatory, and antioxidant effects; however, their effects on cardiac protection have not been investigated.

**Objective:** The study aims to test the biological activity of *Trapa natans* L. extract (TNE) in cisplatin (CDDP)-induced cardiotoxicity.

**Methods:** Wistar albino rats received a single dose of CDDP intraperitoneally and TNE ones per day for 2 weeks orally. Cardiac inflammation, necrosis, and fibrosis were determined by histological and immunohistochemical analyses. Cytokines in rat sera and cardiac tissue were detected by enzyme-linked immunosorbent assay (ELISA) and quantitative real-time (qRT)-PCR. Rat macrophages cultured in the presence of TNE for 48 h were harvested for flow cytometry, while supernatants were collected for cytokine and reactive oxygen species (ROS) measurement.

**Results:** Application of TNE significantly attenuated CDDP induced cardiotoxicity as demonstrated by biochemical and histopathological analysis. Administration of TNE once daily for 14 days decreased level of proinflammatory (TNF-*α*, IFN-*γ*, and IL-6) and prooxidative parameters (NO_2_, O_2_, and H_2_O_2_), while increased level of immunosuppressive IL-10 and antioxidative glutathione (GSH), catalase (CAT) and uperoxide dismutase (SOD) in the systemic circulation. TNE treatment resulted in attenuated heart inflammation and fibrosis accompanied with the reduced infiltration of macrophages and reduced expression of proinflammatory and profibrotic genes in heart tissue of CDDP-treated animals. In vitro lipopolysaccharide (LPS)-stimulated macrophages cultured in the presence of TNE adopted immunosuppressive phenotype characterized by decreased production of proinflammatory cytokines and prooxidative mediators.

**Conclusion:** Our study provides the evidence that TNE ameliorates cisplatin-induced cardiotoxicity in rats by reducing inflammation and oxidative stress via promoting M2 macrophage polarization.

## 1. Introduction

One of the most effective anticancer drugs used for the treatment of various malignancies is cisplatin (cis-diamminedichloroplatinum [II], CDDP). However, the clinical use of this platinum-based antineoplastic drug is markedly limited by the risk of cardiotoxicity, which can manifest by arrhythmias, atrial fibrillation, angina pectoris, congestive heart failure, myocarditis, and abrupt cardiac death [1, 2]. Inflammatory response, oxidative stress, apoptosis, and necrosis are the central toxic mechanisms in the pathophysiology of CDDP-induced cardiotoxicity [2, 3]. Existing strategies aimed at the prevention and treatment of cardiovascular complications that occur during chemotherapy treatment are focused on typical cardiovascular drugs, which uphold increased cardiac reserve and reverse myocardial remodeling [4]. However, these treatments do not modulate complex and intricate mechanisms and downstream mediators of CDDP-induced cardiac toxicity. Accordingly, an urgent demand exists for researchers to develop new adjuvants for the attenuation of cardiotoxic side effects following cancer treatment in order to improve the survival rate and quality of life of cancer patients Recent studies suggest that herbal formulations and plant-derived products can be successfully used in order to prevent, control, or block cardiovascular disease progression by multitarget action [5]. Thus, natural products have become a research hotspot in medical applications due to their rich antioxidant and anti-inflammatory potential.

*Trapa natans* L. (family: *Trapaceae*), commonly known as the water chestnut, is a widespread annual water plant with floating rosettes of leaves [6]. The chemical composition of water chestnut fruits and leaf extracts has drawn significant attention from scientists and clinicians because of their wide range of health-beneficial properties. *T. natans* L. contains significant amounts of organic and inorganic constituents, vitamins, and dietary fibers [7–9]. The most important substances in the *Trapa natans* L. extract (TNE) are phenolics, including phenolic acids, gallic, ellagic, and ferulic acid, as well as quercetin 3-O-galactoside (hyperoside). The high phenolic content of TNE contributes to their immunomodulatory, anti-inflammatory, antioxidant, anticancer, analgesic, anti-irritation, antiulcer, antidiabetic, and hepatoprotective effects [7–13]. Our earlier studies showed the safety of the *T. natans* leaf extracts for human use as well as the antimicrobial potential against medically important pathogens [7]. Plant extracts of the species *Trapa* decrease the intracellular accumulation of reactive oxygen species (ROS) and reduce proinflammatory mediators through suppression of the NF-*α*B-mediated signaling pathways [10, 12]. Antihyperglycemic and hepatoprotective effects of the examined TNE and its derived fractions have been shown in the streptozotocin-induced diabetic rat model [14]. In particular, antidiabetic compounds isolated from *T. natans* L. increased GLUT4 protein expression in C2C12 myotubes and increased the phosphorylation of the main signaling pathways for glucose uptake regulation, AMPK and AKT[11].

The inhibition of inflammation and oxidative damage is thus a crucial property of TNE, by virtue of which they can inhibit the induction/progression of a number of diseases implicating these pathophysiological mechanisms. However, to the best of our knowledge, the cardioprotective effects of TNE are still unknown. In addition to various biological effects, the main advantages of TNE in cardiac protection compared to other natural products would be cost effectiveness, ease of availability, freeze–thaw properties, and chemical stability which could be used for pharmaceutical preparations. Here, we explored molecular and cellular mechanisms involved in cardioprotective effects of *T. natans* L. methanol leaf extract against CDDP-induced cardiotoxicity in rats in order to pave the way for new therapeutic approaches that can inhibit or minimize CDDP-mediated cardiac injury.

## 2. Materials and Methods

### 2.1. Plant Extraction and Standardization

The floating leaves of plant *T. natans* L. were collected in Medjuvrsje Reservoir, Central Serbia, and the dried plant material was prepared, as we previously described in our paper [6]. Dried, ground plant material was extracted by maceration using methanol. Stock solutions of extracts (100 mg/mL, w/v) were prepared in DMSO (100%) (Fisher Chemical, Waltham, MA, USA) and stored in the dark at 4°C.

### 2.2. Experimental Design

Male Wistar albino rats (8–10 weeks old, 250–300 g body weight) were used in this study. Animals (obtained from Military Medical Academy, Serbia) were maintained under well-controlled environmental conditions of temperature (23 ± 1°C) and light (12/12 h light/dark cycle) and had free access to food and water.

**Figure 1:**
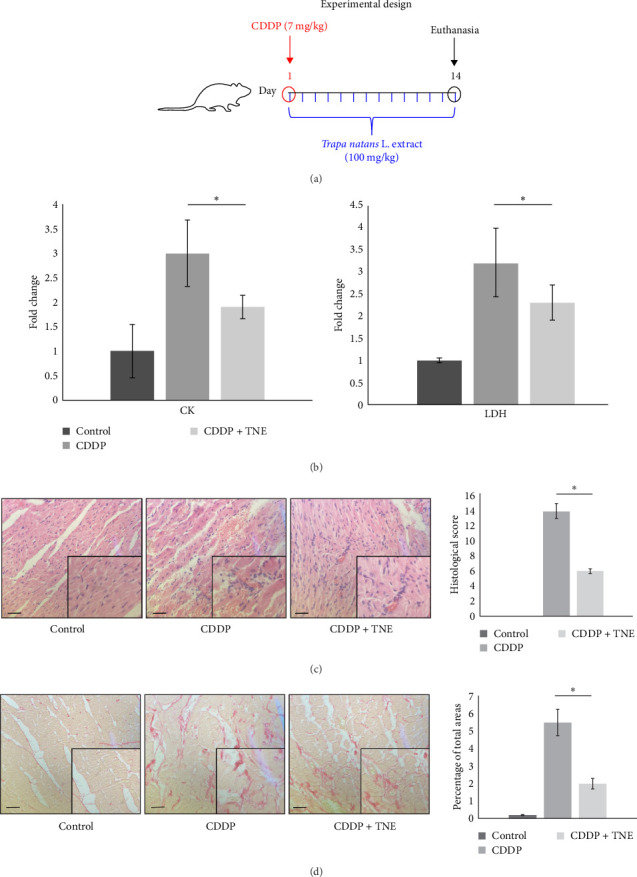
*T. natans* L. extract ameliorates cisplatin-induced cardiotoxicity. (A) Experimental design. Rats were injected with saline, single dose of CDDP (7 mg/kg/w, *i.p*.), or CDDP (7 mg/kg/w, *i.p*.), and TNE (100 mg/kg/w, *per os* ones per day for 2 weeks). The sera for CK/LDH measurement and hearts for histological analysis were collected 14 days after treatment. (B) Fold change of CK and LDH levels was significantly lower in CDDP + TNE-treated rats compared to rats that received CDDP only. (C) H&E staining of heart tissue sections of CDDP + TNE-treated animals revealed reduced myocytes degeneration, inflammatory cells infiltration, hemorrhage, and vascular congestion compared to CDDP-only treated rats. (D) Sirius red staining of heart tissues obtained from CDDP-treated rats revealed extensive collagen deposition. Stained area of fibrous dense tissue was reduced in CDDP + TNE-treated animals and absent in control animals (magnification x40). Data presented as mean ± SD; *n* = 5 rats per groups. *⁣*^*∗*^*p* < 0.05. CDDP, cis-diamminedichloroplatinum [II]; TNE, *Trapa natans* L. extract.

Cisplatin was purchased from Sigma–Aldrich Co. (St. Louis, MO). It was dissolved in sterilized normal saline and prepared immediately before the treatment. Rats were randomly divided into three groups (each group containing 8 rats): control, CDDP, and CDDP + TNE groups. The CDDP group received a single intraperitoneal injection dose of cisplatin (7 mg/kg body weight) on test day 1[15], while the CDDP + TNE group received a single dose of cisplatin (7 mg/kg, intraperitoneally) on test day 1 and then treated with TNE (100 mg/kg body weight, orally) ones per day for 2 weeks. The control group was injected only with a single dose of saline (intraperitoneally, on test day 1) (Figure 1A). The body weight of each rat was recorded at the beginning and at the end of the experiment. On day 14 after cisplatin administration, animals were sacrificed by decapitation after exposure to ether in a dessicator kept in a well-functioning hood, and tissue samples were obtained for further analyses. All experiments were performed according to EU Directive for Welfare of Laboratory Animals (86/609/EEC) and principles of Good Laboratory Practice (GLP) approved by the Ethical Committee of the Faculty of Medical Sciences, University of Kragujevac, Serbia (approval number 01-16176/1, 16.12.2019).

### 2.3. Biochemical Assays

The samples for determination of biochemical markers of myocardial injury (CK and LDH) were collected immediately after decapitation. A freshly separated sera for estimation of CK and LDH levels in blood was obtained after centrifugation at 3000 rpm for 10 min and preserved at −20°C until the determination of CK and LDH levels. Levels of CK and LDH in serum were determined spectrophotometrically using the Roche Cobas system according to the manufacturer's protocol [16]. The values are presented as U/L.

### 2.4. Histological Analyses

Heart specimens from each group were removed to be examined histopathologically. Heart tissues were fixed in 10% phosphate-buffered formalin, sectioned at 5 µm and stained with hematoxylin and eosin (H&E) stain (Sigma–Aldrich, USA) for light microscopic examination to evaluate the following parameters: (a) disruption of cardiac muscle architecture; (b) loss of muscular striations; (c) myocyte degeneration; (d) fibrosis; and (e) inflammatory cellular infiltrate. Scores were expressed as follows: (0) normal; (1) mild; (2) moderate; and (3) severe[17]. For evaluating the severity of heart damage, a histology scoring system was used, and microscopy was performed in blind fashion by two investigators (V.M. and M.G.J.).

### 2.5. Measurement of Cardiac Fibrosis

Interstitial collagen was visualized using picrosirius red staining, as previously described [18]. Isolated perfused hearts were fixed in 10% buffered formalin, embedded in paraffin, and sliced into a horizontal 5 μm section for sirius red staining (Direct Red 80, Sigma–Aldrich) according to the manufacturer's protocol. The fibrotic area in each sample was analyzed in blind fashion by two investigators (V.M. and M.G.J.). Quantification of fibrosis in mouse heart sections stained with PicroSirius red (10x) was performed using ImageJ software (National Institutes of Health, Bethesda, MD, USA), on 10 fields/section, as described [19].

### 2.6. Measurement of Cytokine Levels

Levels of TNF-*α*, IL-6, IFN-*γ*, and IL-10 in supernatant and rat sera were determined using commercially available enzyme-linked immunosorbent assay (ELISA) kits specific for the rat cytokines (R&D Systems, Minneapolis, MN, USA) according to the manufacturer's instructions [20].

### 2.7. Determination of Oxidative Stress Parameters in Plasma and Erythrocytes

Whole blood samples were collected from each animal after the sacrifice. The concentrations of prooxidative markers, including hydrogen peroxide (H_2_O_2_), superoxide anion radical (O_2_^−^), and nitrites (NO_2_^−^) were measured in plasma samples. Additionally, the concentrations of enzymatic defense system components, catalase (CAT), and superoxide dismutase (SOD), as well as the activity of nonenzymatic antioxidants, such as reduced glutathione (GSH), were determined in the erythrocyte lysate. All mentioned parameters were measured at the appropriate wavelengths spectrophotometrically (UV 1800; Shimadzu Corporation, Kyoto, Japan), according to previously described methods [21].

### 2.8. Quantitative Real-Time-PCR (qRT-PCR)

Total RNA was isolated from heart tissue using TRIzol Reagent (Thermo Fisher Scientific), according to the manufacturer's protocol. Complementary DNA (cDNA) was synthesized using high-capacity cDNA reverse transcription kit (Thermo Fisher Scientific) from 1.5 μg of total RNA, according to the manufacturer's instructions. qRT-PCR was performed using Power SYBR Green PCR Master Mix (Applied Biosystems) and selected primers (Table 1). All reactions were carried out with an initial preincubation period (3 min at 93°C), followed by 40 cycles of 1 min at 93°C, 1 min at 55°C, and 1 min at 72°C. Data were normalized to *β*-actin expression values as a “housekeeping” gene [22].

### 2.9. Immunohistochemical Analysis

For the immunohistochemical staining, we used paraffin-embedded heart sections (5 μm). Deparaffinized tissue sections were incubated with primary antirat CD68 (MS 397-PO; Thermo Fisher Scientific) and primary antirat anti-iNOS (RB-9242-P, Thermo Fisher Scientific), followed by visualization using the HRP/DAB detection IHC Kit (ab236466, Abcam), and sections were counterstained with Mayer's hematoxylin [21]. Sections were photomicrographed with a digital camera mounted on a light microscope (Olympus BX51, Japan), digitized, and analyzed. Analysis was performed on 10 fields/section (magnification ×200). Results are presented as the mean number of positive cells per field.

### 2.10. Isolation and In Vitro Stimulation of Peritoneal Macrophages

Rats were injected with 5 mL of phosphate buffered saline (PBS, Invitrogen) intraperitoneally, and, after shaking, peritoneal lavage was performed. Macrophages were collected from the peritoneal cavity of rats under sterile conditions and cultured in complete Dulbecco's modified eagle medium (DMEM) supplemented with 10% fetal bovine serum (FBS) at 37°C in a 5% CO_2_ incubator (Sigma–Aldrich, Munich, Germany). Isolated macrophages were plated at a density of 10^6^ cells/well, primed with 10 ng/mL LPSs (lipopolysaccharides from *Escherichia coli* L2880, Sigma–Aldrich) for 2 h, and incubated with TNE (30 μg/mL) for 48 h[23]. After 48 h, supernatants were collected for cytokine and ROS measurement, and macrophages were harvested for flow cytometry.

### 2.11. Flow Cytometry

The phenotype of LPS-stimulated macrophages cultured in the presence or absence of TNE was determined by flow cytometry. Briefly, 1 × 10^6^ cell macrophages were incubated with antirat CD11b conjugated with fluorescein isothiocyanate (FITC; BD Biosciences, Franklin Lakes, NJ). For the intracellular staining, cells were previously stimulated with phorbol myristate acetate (PMA) and ionomycin for 4 h at 37°C with the addition of 1 μg/mL Golgi plug. Following extracellular staining, cells were fixed, permeabilized, and stained for IL-4 and IL-10, conjugated with phycoerythrin (PE; BD Biosciences) [24]. Flow cytometric analysis was conducted on a BD Biosciences FACSCalibur and analyzed by using the Flowing software analysis program.

### 2.12. Measurement of O_2_ and NO Generation in TNE-Treated Macrophages

The superoxide anion radical (O_2_^•−^) level was evaluated by a standardized NBT protocol based on the estimation of the reduction of nitroblue tetrazolium (Acros Organics, BP108-1) to nitroblue formazan and the measurement of the purple crystals' absorbance intensity at 630 nm. Macrophages were seeded into 96-well plates and incubated in controlled conditions for 24 h. After the incubation period, the cells were treated with TNE for 24 h. The procedure was comprised of replacing the DMEM containing TNE with 100 μL of fresh DMEM and 10 μL of NBT salt solution (2.5 mg/mL in PBS). After 3 h, the cells were washed of the extracellular O_2_^•−^ with warm PBS and by methanol, followed by adding 100 μL of 2 M KOH for the cell membrane disruption at room temperature. After 15 min, 100 μL DMSO was added for formazan crystal dissolution and absorbance measurement [25].

NO release from TNE-treated macrophages was analyzed by measuring the concentration of nitrite (NO_2_^−^), a hydrolysis product of oxidized NO, in the culture supernatant using the Griess method. The diazotization reaction of nitrites with sulfanilamide (SA; Karl Roth, 4716-100G) and N-1-naphthyleth-ylenediamine dihydrochloride (NED; Karl Roth, 4342-25G) yielded a stable yellow compound, whose absorbance intensity is measured at 492 nm. Similar to the NBT protocol, macrophages were seeded and treated in the same concentration range. After the treatment incubation period, 50 μL of supernatant was transferred to another well plate with a consequent addition of 50 μL of SA and NED. After 15 min, the absorbance was measured [25].

### 2.13. Statistical Analysis

Results were analyzed using the Student's *t* test. All data in this study were expressed as the mean ± standard deviation (SD). Values of *p*  < 0.05 were considered as statistically significant.

## 3. Results

### 3.1. Application of TNE Significantly Attenuates CDDP-Induced Cardiotoxicity

CDDP caused significant cardiac injury as determined by biochemical analysis and histological examination. As shown in Figure 1A, CDDP administration resulted in a threefold increase in CK and LDH when compared to control rats. Application of TNE significantly downregulated serum levels of both CK (*p*  < 0.05) and LDH (*p*  < 0.05) in CDDP-treated animals, suggesting beneficent effects of TNE in the treatment of CDDP-induced cardiotoxicity.

In the control group, the heart tissue was of normal histological appearance, showing regular bundles of muscle fibers and homogenous acidophilic cytoplasm. In agreement with previous studies, heart sections of CDDP-treated animals showed wide areas of myocyte degeneration replaced by fibroblasts, mononuclear cell infiltration between the muscle fibers, vascular occlusion, and separation of myocytes by extravasated red blood cells with loss of muscular striations (Figure 1B). In contrast, histological analysis indicated that TNE-treated rats were less sensitive to CDDP-induced cardiac injury. Heart tissue sections of CDDP + TNE-treated animals showed a significant reduction in heart injury characterized by reduced infiltration of inflammatory cells and small-vessel occlusion (Figure 1B). The damage score calculated according to histopathological findings increased significantly in the CDDP group compared to the control and confirmed a significant reduction of cardiac injury in CDDP-treated rats that received TNE (Figure 1B).

Sirius red staining of heart tissues obtained from CDDP-treated rats revealed extensive collagen deposition, suggesting the development of cardiac fibrosis. TNE administration significantly decreased the fibrotic size and the interstitial collagen expression in the perivascular region in a CDDP-induced cardiotoxicity model (Figure 1C).

### 3.2. TNE Altered Serum Levels of Cytokines That Play a Crucial Role in CDDP-Induced Cardiotoxicity

**Figure 2:**
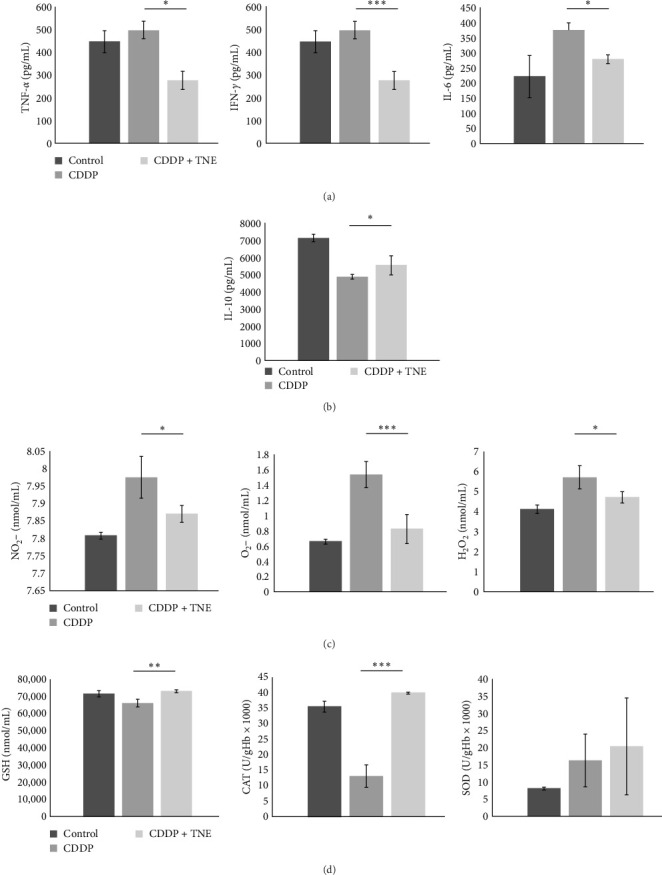
*T. natans* L. extract attenuates inflammation and oxidative damage in CDDP-treated rats. (A) The concentration of proinflammatory TNF-*α*, IFN-*γ*, and IL-6 were significantly decreased and (B) level of anti-inflammatory IL-10 was significantly increased in serum of CDDP + TNE-treated animals compared to rats that received CDDP only. (C) Levels of prooxidative parameters (NO_2_ˉ, O_2_ˉ, and H_2_O_2_) were significantly lower, while (D) levels of antioxidative parameters (GSH, CAT, and SOD) were significantly higher in systemic circulation of CDDP + TNE-treated animals compared to rats that received CDDP only. Data presented as mean ± SD; *n* = 5 rats per groups. *⁣*^*∗*^*p* < 0.05, *⁣*^*∗∗*^*p* < 0.01, *⁣*^*∗∗∗*^*p* < 0.001. CDDP, cis-diamminedichloroplatinum [II]; TNE, *Trapa natans* L. extract.

In order to explore whether cardioprotective effects of TNE are a consequence of their effects on systemic immune response, cytokine concentration was determined in the sera of the experimental animals. In accordance with the biochemical and histological analysis, TNE significantly attenuated the production of inflammatory cytokines. The concentrations of TNF-*α*, IFN-*γ*, and IL-6 cytokines were significantly lower (TNF-*α*, *p*  < 0.05; IFN-*γ*, *p*  < 0.001; IL-6, *p*  < 0.001; Figure 2A) while the concentration of anti-inflammatory IL-10 was significantly higher (*p*  < 0.05) in sera of CDDP-treated rats that received TNE (Figure 2B).

### 3.3. The Levels of Prooxidative and Antioxidative Parameters in Systemic Circulation

We further investigated the effects of TNE on the concentration of oxidative stress biomarkers in the systemic circulation of CDDP-treated animals. CDDP resulted in a significant increase in oxidative parameters and a significant decrease in antioxidative parameters as compared to the control group (Figure 2C). However, administration of TNE for 14 consecutive days after a single dose of CDDP showed a significant decrease in oxidative biomarkers NO_2_, O_2_, and H_2_O_2_ (NO_2_, *p*  < 0.05; O_2_, *p*  < 0.05; H_2_O_2_*p*  < 0.001; Figure 2C). Moreover, the CDDP + TNE-treated group presented significantly higher values of the antioxidative parameters GSH and CAT (GSH, *p*  < 0.01; CAT, *p*  < 0.001; Figure 2D) compared to the CDDP group. The difference in the concentration of antioxidative SOD did not reach statistical significance (*p*  > 0.05) between groups.

### 3.4. TNE Modulates Expression of Inflammation- and Fibrosis-Related Genes in the Heart

**Figure 3:**
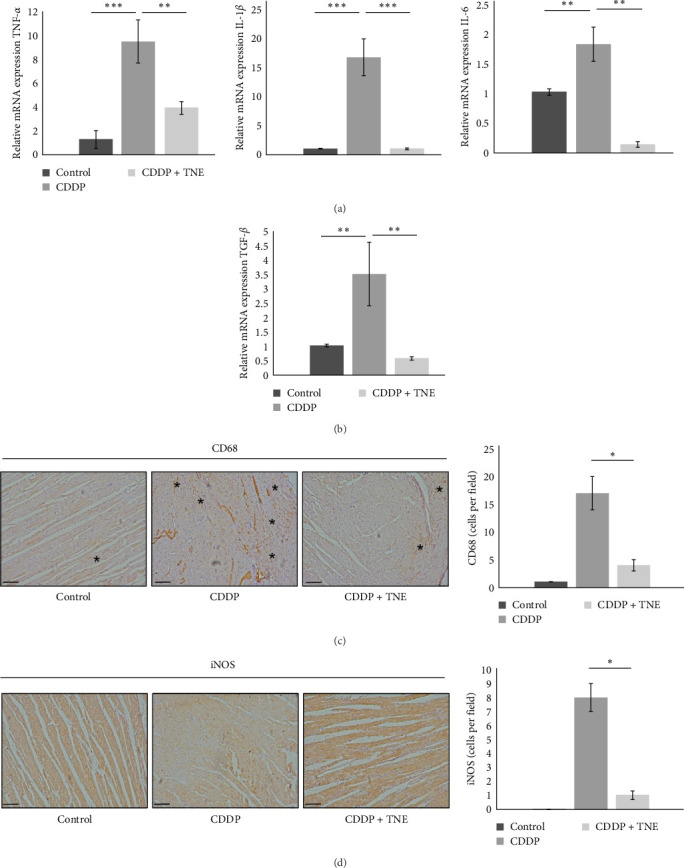
Application of *T. natans* L. extract reduces the presence of macrophages and inflammatory cytokines in the hearts of CDDP-treated rats. TNE decreased relative expression of (A) proinflammatory cytokine genes (TNF-*α*, IL-1*β*, and IL-6) as well as (B) profibrogenic marker TGF-*β* in the heart tissue of CDDP-treated rats. Representative immunohistochemical images and number of (C) CD68^+^ macrophages and (D) iNOS^+^ cells per field in the paraffin-embedded heart tissue sections (×20). Data presented as mean ± SD; *n* = 5 rats per groups. *⁣*^*∗*^*p* < 0.05, *⁣*^*∗∗*^*p* < 0.01, *⁣*^*∗∗∗*^*p* < 0.001. CDDP, cis-diamminedichloroplatinum [II]; TNE, *Trapa natans* L. extract.

Attenuated heart damage, noticed in CDDP + TNE-treated rats, correlated with the expression of major inflammation-related genes in CDDP-mediated cardiotoxicity. TNE significantly reduced (*p*  < 0.05) the expression of mRNA encoding proinflammatory TNF*α*, IL−1*β*, and IL−6 in the cardiac tissue of CDDP-treated rats (Figure 3A). In accordance with the histological staining of collagen (Figure 1C), qRT-PCR showed that TNE treatment significantly decreased the expression of TGF-*β* (*p*  < 0.05) known as a major profibrogenic cytokine associated with organ fibrogenesis (Figure 3B) [26, 27].

### 3.5. TNE Reduced Infiltration of Macrophages in the Heart of CDDP-Treated Rats and Attenuated Expression of iNOS in Cardiac Tissue

In order to dissect out the cellular target of TNE-dependent modulation of CDDP-induced cardiotoxicity, we next investigated the composition of the inflammatory infiltrate on the longitudinal sections of the heart tissue using immunohistochemistry. As shown in Figure 3, a single injection of CDDP significantly increased the influx of inflammatory CD68^+^ macrophages and the expression of iNOS in the cardiac tissue of rats compared with the control group (Figure 3). However, intracellular staining revealed that there were significantly lower numbers of CD68^+^ cardiac-infiltrating macrophages in the heart of CDDP + TNE-treated rats when compared with CDDP-only treated animals (Figure 3C). Moreover, application of TNE significantly reduced the mean number of iNOS^+^ inflammatory cells in the heart tissue samples of the CDDP-treated group (Figure 3D).

### 3.6. TNE Reduces Inflammation and Oxidative Stress in Rat Macrophages

**Figure 4:**
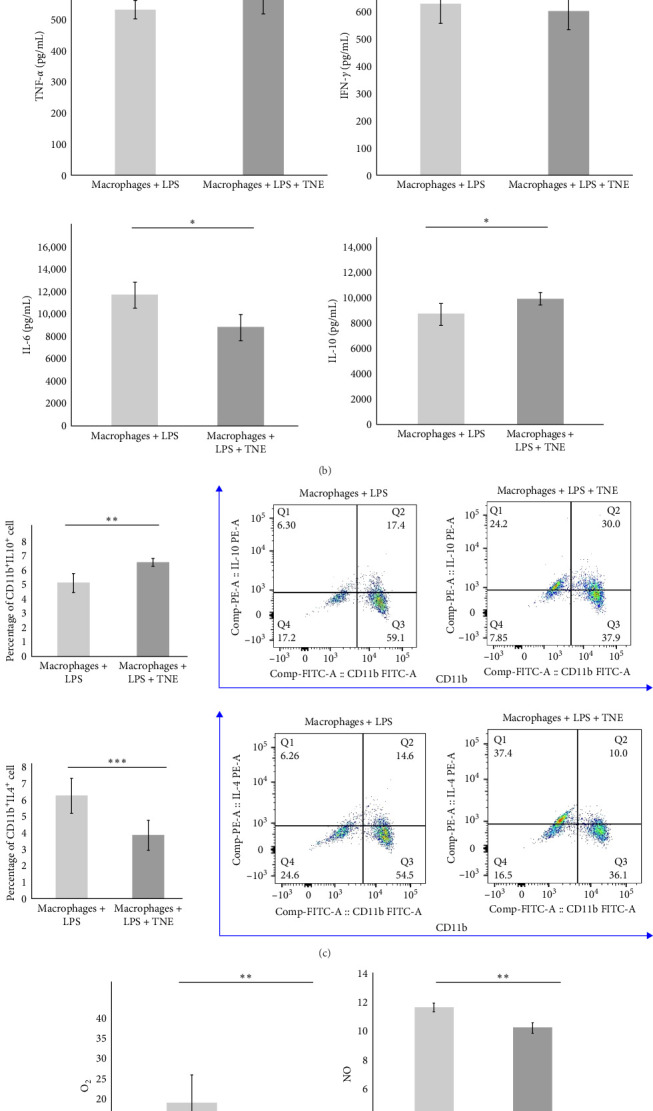
TNE suppress reactive oxygen species and proinflammatory cytokine responses of macrophages. (A) Schematic diagram describing design of in vitro experiments. In vitro LPS-stimulated peritoneal macrophages isolated from healthy rats were cultured for 48 h with or without TNE. Phenotype of peritoneal macrophages, as well as level of cytokines and prooxidative parameters in supernatants, was analyzed. (B) The significantly lower amounts of IL-6 and higher amount of IL-10 were noticed in supernatants of in vitro LPS-stimulated peritoneal macrophages cultured with TNE. Difference in the concentration of TNF-*α* and IFN-*γ* in the supernatant did not reach statistical difference. (C) Flow cytometry revealed significantly higher percentage of IL-10-producing-, and lower percentage of IL-4-producing LPS-activated CD11b+ macrophages cultured in the presence of TNE, when compared to LPS-stimulated macrophages that were cultured without TNE. (D) TNE-induced significant decrease in concentration of prooxidative parameters O2 and NO in the supernatant of LPS-stimulated CD11b+ macrophages. *⁣*^*∗*^*p* < 0.05, *⁣*^*∗∗*^*p* < 0.01, *⁣*^*∗∗∗*^*p* < 0.001. TNE, *Trapa natans* L. extract.

In order to further investigate the immunomodulatory effects of TNE on the major effector cells in the rat model of CDDP-induced cardiotoxicity, LPS-stimulated macrophages were cultured with or without TNE (Figure 4A). In accordance with data obtained in the animal model, a significantly lower (*p*  < 0.05) concentration of cardiotoxic and inflammatory cytokine IL-6 was noticed in the supernatants of in vitro stimulated macrophages cultured in the presence of TNE (Figure 4B). There was no difference in the levels of TNF-*α* as well as IFN-*γ* between groups (Figure 4B). However, levels of anti-inflammatory IL-10 in the supernatants derived from macrophages stimulated in the presence of TNE were significantly higher (*p*  < 0.05) (Figure 4B). Flow cytometry staining confirmed a significantly lower percentage of inflammatory IL-4-producing CD11b+ cells, cultured in the medium that contained TNE, when compared to cells that were cultured without TNE (*p*  < 0.001, Figure 4C). Moreover, incubation with TNE induced a significant increase in the percentage of protective IL-10-producing CD11b+ macrophages (*p*  < 0.01, Figure 4C), suggesting an anti-inflammatory effect of TNE. These results indicate that TNE reduced the inflammatory response, specifically promoting M2 rather than M1 differentiation.

Furthermore, incubation with TNE induced a significant inhibition of oxidative stress in LPS-stimulated macrophages. Release of both, O_2_ and NO, from LPS-stimulated macrophages was significantly (O_2_, *p* <0.01 and NO, *p*  < 0.001) reduced in the presence of TNE (Figure 4D).

## 4. Discussion

Cisplatin has been used as cancer chemotherapeutics for the last several decades[28]. However, cisplatin-based chemotherapy has been reported to cause cardiovascular diseases, particularly myocardial infarction and angina, in a range of 7%–32% of patients [29, 30]. Much effort has been put into the development of new adjuvant therapies, but none of them has reached worldwide clinical application so far. Most recently, we identified and characterized 22 phenolic compounds in TNE, where 13 of them were identified in *T. natans* L. for the first time [7]. Although the biological activity of TNE has been reported, the cardioprotective potential of TNE in induction and progression of CDDP-caused cardiac injury is still unknown. Here, we provide the first evidence that TNE alleviates CDDP-induced cardiotoxicity by attenuating cardiac inflammation, fibrosis, and oxidative damage through suppression of macrophage infiltration and activation.

Although the precise mechanisms are not defined, various studies have confirmed that both oxidative stress and inflammation exert cytotoxic effects in the context of CDDP-mediated cardiotoxicity [31–33], leading to cardiac remodeling [34], and extensive degeneration and fragmentation of cardiac muscle fibers [33]. Disruption of cell membranes caused by cisplatin administration leads to a release of intracellular proteins, such as dehydrogenase, creatine kinase, and troponin [16]. We showed that TNE administration significantly reduced necrosis, mononuclear cell infiltration, fibrosis, and hemorrhage in cardiac tissue of CDDP-treated rats that were accompanied with lower serum levels of myocardial enzymes (Figure 1).

Cisplatin-induced cardiac toxicity is primarily based on disruption of the redox balance in the cells, due to depletion of the antioxidant glutathione and impairment of oxidative phosphorylation, leading to increased generation of ROS in the damaged mitochondria, including superoxide anions, H_2_O_2_, and hydroxyl radicals [35, 36]. Cellular effects of ROS are enhanced by the production of NO, mediated by the inducible NO synthase, which leads to the continuous generation of peroxynitrite, which further reacts with O^2−^• contributing to tissue damage by cisplatin [37]. The overproduced oxidative radicals after CDDP administration react with cell membrane lipids, proteins, and nucleic acids, resulting in cardiomyocyte necrosis and apoptosis [38]. El-Awady et al. [39] confirmed that glutathione and SOD levels were reduced in cardiac tissue of cisplatin-treated rats [39]. Thus, increasing antioxidant levels in the heart through exogenous administration of antioxidants could reduce the ROS production in cardiac tissue and may help protect the heart against cisplatin toxicity [39]. A previous study indicates that bioactive components of examined aqueous extract of *T. natans* L. fruits, scavenged O_2_, H_2_O_2_, and NO in vitro effectively and dose dependently and provided protection against oxidative damage [12]. We have already demonstrated that the most important substances in the aqueous fruit extract of *T. natans* L. were phenolics with strong antifree radical properties [7]. Consistent with previous experimental data, here we found that TNE had significant in vivo antioxidant action against free radicals (Figure 1). TNE ameliorated the CDDP-induced cardiac damage by decreasing the levels of oxidative biomarkers NO_2_, O_2_, and H_2_O_2_ and increasing the values of antioxidative parameters CAT and GSH (Figure 2C,D). Elevated CAT preserves cardiac morphology and contractile function, mitigates inflammation, and prevents the development of myocyte hypertrophy, apoptosis, and interstitial fibrosis [40]. Increased GSH efficiently scavenges ROS and nitrogen species, either directly or indirectly, by acting as a cofactor to support the activity of various enzymes [41].

Due to impaired oxidative status, CDDP intoxication significantly increases the level of the proinflammatory mediators via NF-*κ*B pathway activation in the cardiac tissue [34], leading to the excessive accumulation and activation of immune cells in the heart and severe cardiac tissue injury [32]. Moreover, it is also known that cisplatin-induced cellular damage and necrosis lead to the release of damage-associated molecular patterns, which activate the innate immune system by interacting with pattern-recognition receptors [36]. Among the immune cells, macrophages play a central role in the propagation of the inflammation and induction of cell apoptosis after muscle injury [42]. Sung et al. [42] found that ablation of tissue-resident macrophages represents a novel strategy for mitigating cisplatin-induced ototoxicity and nephrotoxicity. TNE administration suppressed oxidative stress-associated inflammation and improved cardiac function by inhibiting CDDP-induced cardiac influx of macrophages and down-regulating iNOS expression in cardiac tissue (Figure 3). Macrophage-dependent CDDP toxicity is associated with increased production of ROS generated through NADPH oxidase 3 as well as secretion of proinflammatory cytokines, such as TNF*α*, IL-1*β*, and IL-6 through activation of NF-*κ*B, a transcription factor and regulator of innate immunity [42]. M1 macrophages may also contribute to cisplatin-induced toxicity by producing NO as an important proinflammatory cytotoxic agent. We found that a significantly lower number of cardiac-infiltrated macrophages was accompanied by decreased expression of inflammatory mediators TNF*α*, IL-1*β*, and IL-6 in the cardiac tissue (Figure 1D). Consequently, there were lower serum levels of inflammatory cytokines and a higher serum concentration of cardioprotective IL-10 in CDDP + TNE-treated rats (Figure 2A, B). By producing IL-10, alternatively, activated M2 macrophages have been known to repress the excessive inflammatory response and promote myogenesis [43]. Thus, regulating the balance of M1 and M2 macrophages in cardiac tissue to prevent the progression of inflammation can be considered as a promising therapeutic strategy for CDDP-induced toxicity.

**Figure 5:**
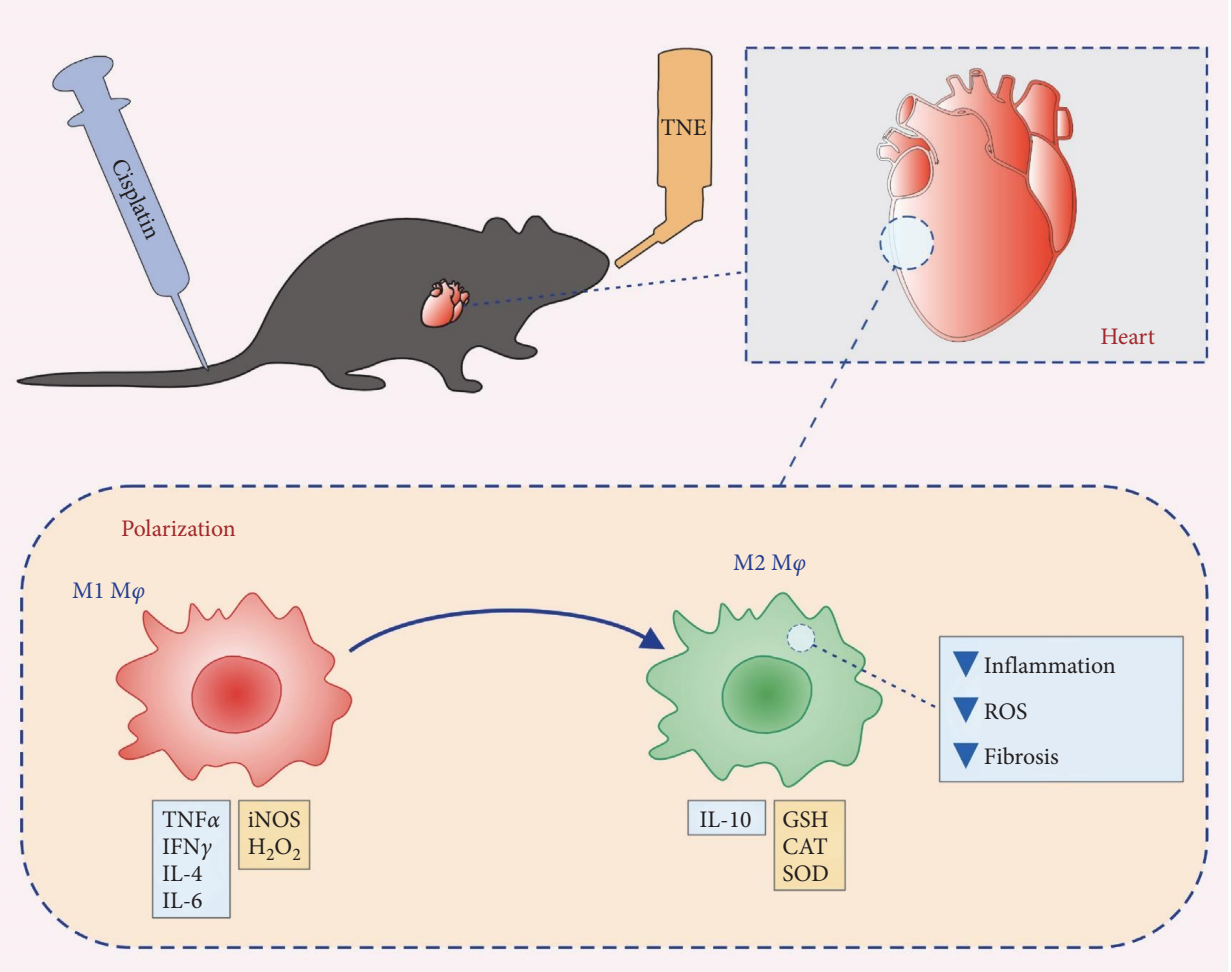
Proposed mechanism of TNE-based attenuation of cisplatin-induced cardiotoxicity. After cisplatin-induced cardiac injury, application of TNE induces polarization of proinflammatory M1 macrophages to the anti-inflammatory and proregenerative M2 phenotype characterized by increased production of protective IL-10 and antioxidative GSH, CAT, and SOD, resulting with attenuated inflammation, oxidative damage, and fibrosis in the heart. CAT, catalase; GSH, glutathione; SOD, superoxide dismutase.

To elucidate the underlying mechanisms by which macrophage inhibition by TNE confers protection against CDDP-induced cardiotoxicity, we examined whether TNE alters the phenotype and function of macrophages in vitro. Intracellular staining showed that there were significantly higher numbers of IL-10-producing alternatively activated M2 macrophages and significantly reduced numbers of IL-4 producing inflammatory CD11b+ macrophages in the presence of TNE (Figure 4C). In line with these findings, significantly higher concentration of anti-inflammatory IL-10 and lower concentration of inflammatory IL-6 (Figure 4B) were noticed in the supernatant of LPS-stimulated macrophages that were cultured with TNE, confirming the capacity of TNE to induce macrophage polarization into the anti-inflammatory M2 phenotype. Moreover, peritoneal macrophages were found to produce high amounts of NO and O_2_ after stimulation with LPS, an effect that was reversed after treatment with TNE (Figure 4D). Having in mind that M1 macrophages express numerous cytokines, reactive nitrogen, and oxygen intermediates, which have a strong proinflammatory activity, while the M2 phenotype expresses molecules including IL-10, which are supposed to be involved in tissue remodeling and immunoregulatory functions [44], here we propose that targeting macrophage polarization and skewing their phenotype by TNE administration might hold great promise for the treatment of CDPP-mediated cytotoxicity (Figure 5).

After cardiac injury, alterations in extracellular matrix (ECM) homeostasis, and the release of growth factors and inflammatory cytokines by immune cells dynamically modulate cardiac fibroblast characteristics and functions, leading to myocardial fibrosis [45, 46]. TGF-*β* is a major profibrotic mediator that stimulates cardiac inflammation and fibrosis through activation of NF-*κ*B signaling [47–49]. This multifunctional cytokine not only activates fibroblasts and stimulates them to differentiate into myofibroblasts, which produce ECM proteins in the heart muscle but also promotes fibroblast proliferation [50]. Accumulating evidence indicates that reduction of inflammatory factors and/or inhibition of oxidative stress can significantly reduce TGF*β*-mediated cardiac fibrosis in different heart diseases [51, 52]. TNE attenuates cardiac fibrosis in CDDP-treated rats by inhibiting the expression of inflammation-related factors (TNF*α*, IL-1*β*, and IL-6) and suppressing macrophage influx and secretion of TGF*β*. Moreover, levels of metabolic products associated with the oxidative stress and positively correlated with severity of cardiac fibrosis are also decreased in TNE-treated animals. TNE administration significantly inhibited TGF-*β* expression and also inhibited the profibrotic effects of TGF-*β* signaling via indirect mechanisms, leading to reduced collagen deposition in the heart of CDDP-treated rats (Figure 1D).

Our data suggest that TNE can protect against cardiotoxicity induced by CDDP. The protective effect of TNE is associated with attenuation of necrosis, fibrosis, production of inflammatory mediators, and intracardiac macrophage recruitment, shifting the balance between pro- and anti-oxidative enzymes in favor of antioxidative, and by polarizing macrophages to the anti-inflammatory and pro-regenerative M2 phenotype (Figure 5). Consequently, our findings highlight the TNE as a potential therapeutic agent that could have cardioprotective effects.

The obvious advantages of this natural product over most other strategies used for treatment of cardiovascular complications that occur during chemotherapy are economical production, stability, and ease of oral administration. Additionally, TNE might be a considerable adjuvant therapy in oncology due to its confirmed anticancer properties. However, cardioprotective characteristics of TNE should be thoroughly analyzed and considered in future clinical trials in order to avoid potential undesirable interactions with the immunomodulatory drugs that are used in cancer treatment.

The strength of the present study is that for the first time, the cardioprotective effects of the *T. natans* L. methanol leaf extract are reported. However, the limitations of the study are given by the animal species (only one has been used) and the short-term evaluation. To overcome these limitations for future perspectives, replication of this investigation using different in vivo models would improve the strength of the analysis. Second, since we suggest that TNE has therapeutic potential in CDDP-induced cardiotoxicity, it is important to explore and assess cardiac injury and outcome in a longer follow-up.

## 5. Conclusions

Based on these findings, it could be concluded that TNE attenuates inflammation, oxidative damage, and fibrosis in CDDP induced cardiotoxicity by promoting the polarization of macrophages from proinflammatory M1 to anti-inflammatory M2 phenotype. TNE downregulates production of proinflammatory and prooxidative markers (such as TNF-*α*, IFN- *γ*, IL-6, ROS, and NO) by M1 macrophages and increases production of anti-inflammatory and repair-promoting mediators (IL-10, GSH, and CAT) related to M2 macrophages, resulting with the beneficial effects in CDDP-induced cardiotoxicity. We assume that TNE may prevent ventricular remodeling and preserve left ventricle ejection fraction, by reducing direct cytotoxic effects of CDDP, such as interruption of myofibrils and severe interstitial hemorrhages or fibrosis. TNE administration could be suitable for alleviating the long-term cardiovascular adverse effects after CDDP treatment including metabolic dysfunction and myocardial ischemia. These findings could be helpful in developing new, TNE-based therapeutic approaches for attenuation of CDDP-induced cardiotoxicity. Our results clearly indicated that in the future, more attention should be paid to the natural active compounds for preventing and treating cardiovascular complications induced by chemotherapeutics.

## Figures and Tables

**Table 1:** Primers used for qRT-PCR analysis.

Gene	Sense and antisense
Rat IL-1*β* primer pair	F: TGATGTTCCCATTAGACAGC R: GAGGTGCTGATGTACCAGTT
Rat TNF-*α* primer pair	F: GTAGCCCACGTCGTAGCAAA R: CCCTTCTCCAGCTGGAAGAC
Rat IL-10 primer pair	F: CAGTCAGCCAGACCCACAT R: GCTCCACTGCCTTGCTTT
Rat TGF-*β*1 primer pair	F: TTTAGGAAGGACCTGGGTTG R: CAGACAGAAGTTGGCATGGTe
Rat *β*-actin primer pair	F: ACGGTCAGGTCATCACTATCG R: GGCATAGAGGTCTTTACGGATG
Rat IL-6 primer pair	F: CTTCCAGCCAGTTGCCTTCT. R: GACAGCATTGGAAGTTGGGG

## Data Availability

The data that support the findings of this study are available from the corresponding author upon reasonable request.
